# Evaluation of Genetically Inactivated Alpha Toxin for Protection in Multiple Mouse Models of *Staphylococcus aureus* Infection

**DOI:** 10.1371/journal.pone.0063040

**Published:** 2013-04-29

**Authors:** Rebecca A. Brady, Christopher P. Mocca, Ranjani Prabhakara, Roger D. Plaut, Mark E. Shirtliff, Tod J. Merkel, Drusilla L. Burns

**Affiliations:** 1 Center for Biologics Evaluation and Research, Food and Drug Administration, Bethesda, Maryland, United States of America; 2 Department of Microbial Pathogenesis, University of Maryland School of Dentistry, Baltimore, Maryland, United States of America; 3 Department of Microbiology and Immunology, University of Maryland School of Medicine, Baltimore, Maryland, United States of America; Instituto Butantan, Brazil

## Abstract

*Staphylococcus aureus* is a major human pathogen and a leading cause of nosocomial and community-acquired infections. Development of a vaccine against this pathogen is an important goal. While *S. aureus* protective antigens have been identified in the literature, the majority have only been tested in a single animal model of disease. We wished to evaluate the ability of one *S. aureus* vaccine antigen to protect in multiple mouse models, thus assessing whether protection in one model translates to protection in other models encompassing the full breadth of infections the pathogen can cause. We chose to focus on genetically inactivated alpha toxin mutant HlaH35L. We evaluated the protection afforded by this antigen in three models of infection using the same vaccine dose, regimen, route of immunization, adjuvant, and challenge strain. When mice were immunized with HlaH35L and challenged via a skin and soft tissue infection model, HlaH35L immunization led to a less severe infection and decreased *S. aureus* levels at the challenge site when compared to controls. Challenge of HlaH35L-immunized mice using a systemic infection model resulted in a limited, but statistically significant decrease in bacterial colonization as compared to that observed with control mice. In contrast, in a prosthetic implant model of chronic biofilm infection, there was no significant difference in bacterial levels when compared to controls. These results demonstrate that vaccines may confer protection against one form of *S. aureus* disease without conferring protection against other disease presentations and thus underscore a significant challenge in *S. aureus* vaccine development.

## Introduction

*Staphylococcus aureus* is a leading cause of both nosocomial and community-acquired infections in the United States [1], [2]. *S. aureus* infections range from superficial skin infections to life-threatening, invasive illnesses such as sepsis, osteomyelitis, and endocarditis. This bacterium colonizes the anterior nares of approximately 28% of the population [3], and infections can be caused by the colonizing strain [4]–[6]. *S. aureus* features an extensive and highly redundant repertoire of virulence factors that allow it to colonize and damage the host, as well as evade host immune responses and cause a variety of diseases in multiple host compartments. The profile of expressed proteins exhibited by this pathogen can vary according to growth state (planktonic vs biofilm) [7]–[9]. Therefore, the potential exists for antigens expressed in one type of infection (e.g., acute infections such as sepsis) to not be present in other types of infection, such as chronic biofilm infections.

*S. aureus* infections are increasingly caused by antibiotic resistant isolates, including the well known methicillin resistant strains (MRSA). In the past decade, vancomycin resistant *S. aureus* (VRSA) has also been isolated, highlighting the ability of the bacterium to adapt to the current repertoire of therapies available and illustrating the importance of a prophylactic vaccine for *S. aureus*. A number of antigens have been tested for their ability to protect animals against various *S. aureus* infections, including adhesins [10]–[21], other surface proteins [22]–[29], secreted toxins [30]–[32], and polysaccharides [33]–[35]. However, to this point, no *S. aureus* vaccine exists.

For an *S. aureus* vaccine to be broadly applicable, it would need to protect against a variety of *S. aureus* infections. Thus far, however, most protective antigens identified in the literature were tested in one or only a few animal models of *S. aureus* disease that do not represent the full breadth of diseases caused by this organism. In this work, we wished to determine the protective capability of a single antigen in multiple models of *S. aureus* disease that encompass a broad spectrum of bacterial growth–from infection with planktonic *S. aureus* to biofilm infection, from acute to chronic disease, and from superficial infection limited to the skin to invasive systemic illness. For each model, we administered the antigen using the same dose, same dosing schedule, and same adjuvant in order to mimic how vaccines are actually formulated and used. We also used the same challenge strain in each model. For this study, we chose to use a genetically inactivated form of alpha hemolysin (HlaH35L) [36] as the vaccine antigen. Immunization with this antigen has previously been shown to decrease mortality and lung colonization in a mouse pneumonia model [32], decrease lesion size and lessen dermonecrosis in a murine skin infection model [30], and also lessen lethality in a peritonitis model [37]. A truncated Hla was very recently shown to elicit protection in both pneumonia and bacteremia models [38]. A heat-inactivated form of alpha-toxoid has also been shown to protect against corneal damage in a *S. aureus* keratitis model [39]. However, these studies used different antigen preparations, different dosing, and different adjuvants, which do not reflect the likely real world scenario in which a single vaccine formulation would be used and administered on a single dosing schedule.

In this study, we tested purified, recombinant HlaH35L in three mouse models of USA300 infection: systemic infection (IV), skin and soft tissue infection (SSTI), and chronic prosthetic implant infection (PII). Utilizing the same antigen, dosing regimen, route of immunization, adjuvant and the same bacterial challenge strain in all three models allowed for evaluation of potential variation in protective capability of the antigen against different disease forms. Results using these three divergent models indicated a differential ability of recombinant HlaH35L to protect against staphylococcal illnesses that are at different ends of the infection spectrum.

## Materials and Methods

### Ethics Statement

For all animal studies, protocols were reviewed and approved by the Institutional Animal Care and Use Committees (IACUC) of the Center for Biologics Evaluation and Research (Bethesda, MD; permit number 2010-04) or the University of Maryland School of Medicine (Baltimore, MD; permit number 09-07-02). All surgeries were performed under ketamine/xylazine anesthesia, and all efforts were made to minimize animal suffering. Animals were sacrificed at the time points indicated below using CO_2_ inhalation.

### Bacterial Strains, Plasmids, and Reagents

*S. aureus* USA300 strain NRS384 (NARSA) was used as a PCR template for cloning. PCR primers were generated by the CBER Core Facility (Bethesda, MD). Chemi-competent TOP10 *Escherichia coli* cells and the pTRChis2c expression plasmid were obtained from Invitrogen (Carlsbad, CA). Chemi-competent BL21 *E. coli* were generated in our laboratory as described by Mandel and Higa [40]. *S. aureus* strain SAP149, which is NRS384 expressing a modified *lux* operon from *Photorhabdus luminescens* on an integrated plasmid (unpublished data), was used for all challenge experiments. All reagents were obtained from ThermoFisher Scientific (Waltham, MA) unless noted.

### Cloning, Expression, and Purification of HlaH35L

In order to generate non-toxic Hla, the gene was reconstructed by joining together PCR fragments of *hla* using BsaI as described by Stemmer and Morris [41]. The CAC codon at position 35 was altered to CTC to change histidine to leucine. The primers used are shown in Table 1, with the codon change indicated in the DSIE primer. A C-terminal six-histidine tag and stop codon were added in frame with the coding sequence. The PCR fragments were digested with BsaI and cloned into pTRChis2c (Invitrogen) downstream of the *lac* operon. Ligated vectors were then transformed into *E. coli* TOP10 cells. Positive transformants were selected in the presence of ampicillin, and mutants were confirmed by sequencing. Plasmids were then transformed into *E. coli* BL21 for gene expression.

**Table 1:** Primers used in this study.

Primer	Sequence (5′→3′)
Hla USOE	TATGGTCTCGGATCCAGCAGATTCTGATATTAACATTAAAACC
Hla USIE	TATGGTCTCGCATGCCATTTTCTTTATCATAAGTG
Hla DSOE	TATGGTCTCCTCGAGTTA*ATGATGATGATGATGATG*ATTTGTCATTTCTTCTTTTTCC
Hla DSIE	TATGGTCTCACATGCTCAAAAAAGTATTTTATAG

USOE: Upstream outer end; USIE: Upstream inner end; DSOE: Downstream outer end; DSIE: Downstream inner end. Upstream primers were used to generate a PCR product, and downstream primers were used separately to generate a second product. The two products were then ligated together. The codon change from histidine to leucine at position 35 is noted in DSIE (underlined). The six-histidine tag is indicated in DSOE (italics).

For production and purification of recombinant HlaH35L, LB cultures were supplemented with 50 µg/ml ampicillin and inoculated 1∶100 with an overnight growth of BL21/HlaH35L. Cultures were incubated at 37°C with shaking to an optical density at 600 nm (OD_600_) of 0.5, and 1 mM IPTG was added. Incubation continued for four hours, at which point bacteria were harvested by centrifugation.

HlaH35L was purified through affinity chromatography. Briefly, bacterial pellets were resuspended in lysis buffer (50 mM NaH_2_PO_4_, 0.5 M NaCl, pH 8.0, supplemented with 1 mg/ml lysozyme) and incubated on ice for 30 minutes. Resuspended pellets were sonicated and cellular debris removed by centrifugation at 4000×*g* for 15 minutes. Lysates were then applied to Ni-NTA columns (His-Pur, ThermoFisher) and incubated on a rotator for 60 minutes. The column was washed four times with wash buffer (50 mM NaH_2_PO_4_, 0.5 M NaCl, 20 mM imidazole, pH 8.0), and recombinant protein was eluted with 50 mM NaH_2_PO_4_, 0.5 M NaCl, 250 mM imidazole, pH 8.0. The eluted protein was concentrated and diafiltered into 20 mM Tris, 50 mM NaCl, 2 mM EDTA, pH 8.0 using Amicon® Ultra 15 ml 10,000 MWCO Centrifugal Filters (ThermoFisher). Purity was confirmed by SDS-PAGE, and protein was quantified using BCA (Pierce, Rockford, IL) following the manufacturer’s instructions. Recombinant HlaH35L was stored at −70°C until use.

### Immunization

For all three challenge studies, animals were immunized in the following manner: Purified, recombinant HlaH35L was thawed and subsequently diafiltered into phosphate-buffered saline (PBS, Quality Biological, Gaithersburg, MD) using Amicon® Ultra 15 ml Centrifugal Filters (10,000 MWCO). HlaH35L was then quantified by BCA and resolved via SDS-PAGE to confirm concentration and purity before adsorption to Alhydrogel® for one hour on ice (Brenntag Biosector, Frederiksund, Denmark; 2 mg/ml final concentration) with added CpG (CBER Core Facility, Bethesda, MD; 150 µg/ml final concentration). Six week old, female mice were immunized subcutaneously in the flank with 20 µg HlaH35L, 200 µg Alhydrogel®, and 15 µg CpG in 100 µl total volume on days zero and 14. Control mice received the same concentration of Alhydrogel® and CpG without HlaH35L. All mice were bled prior to challenge on day 28.

### Enzyme Linked Immunosorbant Assay (ELISA)

Ninety-six well plates (MaxiSorp, ThermoFisher) were coated with 1 µg/ml HlaH35L diluted in PBS by incubating the plates at 4°C overnight. The plates were washed three times with 0.9% NaCl, 0.05% Tween-20 and then blocked with 2% (w/v) nonfat dry milk in PBS for two hours at 37°C. The blocked plates were washed again, and sera from immunized or control mice were diluted serially in dilution buffer (PBS with 2% fetal bovine serum (Invitrogen) and 0.05% Tween-20), and incubated at RT for two hours. After washing, anti-mouse IgG conjugated to horseradish peroxidase (HRP; KPL Inc., Gaithersburg, MD) was diluted in dilution buffer, added to the plates, and incubated at RT for two hours. Plates were washed again and reactivity was analyzed by adding ABTS® ELISA HRP Substrate (KPL, Inc.) and incubating for ten minutes at RT. The reaction was stopped through the addition of ABTS® Stop Solution (KPL), and optical densities were read at 405 nm (OD_405_) on a VersaMax Microplate Reader (Molecular Devices, Sunnyvale, CA). Data were plotted using GraphPad Prism software (Version 5, GraphPad Software, La Jolla, CA) with a 4-parameter logistic (4PL) curve fit of OD (Y axis) versus logarithm of the reciprocal serum dilution (X axis). Titers are defined as the effective dilution that gives 50% of the maximal absorbance (ED_50_) for each sample.

### Skin and Soft Tissue Challenge

On the day of challenge, an overnight growth of *S. aureus* SAP149 was used to inoculate four 50 ml aliquots of Tryptic Soy Broth (TSB; MP Biomedicals, Solon, OH), supplemented with 10 µg/ml chloramphenicol (Sigma Aldrich, St. Louis, MO), at a dilution of 1∶50. The cultures were grown at 37°C with shaking until an OD_600_ of 0.8 was reached, and then centrifuged at 4000×*g* for 15 minutes. All four bacterial pellets were resuspended together in 50 ml PBS, and bacterial counts were obtained using a Petroff-Hausser Counting Chamber (Hausser Scientific Partnership, Horsham, PA). The bacteria were centrifuged again at 4000×*g* for 15 minutes and then resuspended in an appropriate volume of PBS to give a concentration of 1×10^11^ CFU/ml. The bacterial count was confirmed with serial dilution and plating on Tryptic Soy Agar (TSA; MP Biomedicals).

Immunized and control mice were anesthetized with 2 mg Ketamine (Ketaject, Phoenix Pharmaceutical, St. Joseph, MO) and 0.1 mg Xylazine (AnaSed, Akorn, Decatur, IL) administered IP. Images were obtained of each animal’s left ear using a Nikon SMZ 745T dissecting microscope. Left ears were sterilized with 70% ethanol, and then 1×10^9^ CFU of *S. aureus* SAP149 were administered to the left ear of each mouse in a volume of 10 µl, using a Morrow Brown needle as described by Prabhakara *et al.* [42]. Subsets of animals were photographed on days four and seven following challenge and then sacrificed. The left ear was excised, minced, and homogenized in sterile PBS. Serial dilutions were plated on TSA. A second group of mice was imaged serially on days four, seven, and 14 in order to follow the infection progression over time in the same animals. These mice were sacrificed, and ears were harvested and plated, on day 14 post-challenge.

### Intravenous (IV) Challenge

An overnight culture was started using a single hemolytic colony of *S. aureus* SAP149 from a one-day-old 5% blood agar plate (Becton Dickinson, Sparks, MD) containing 10 µg/ml chloramphenicol and incubated at 37°C overnight. On the day of the challenge experiment, TSB containing 10 µg/ml chloramphenicol was inoculated with a 1∶100 dilution of the overnight growth. The culture was incubated at 37°C with shaking until the OD_600_ reached approximately 0.63. The cell concentration calculation is based on previous experimentation in which it was determined that at an OD_600_ of 0.63 the concentration of the growth is approximately 3.66×10^8 ^CFU/ml (data not shown). The cells were then pelleted by centrifugation at 4000×*g* for 10 minutes. The pellet was re-suspended in 1X PBS (Quality Biological) and brought to a concentration of 3×10^7^ CFU/ml. The challenge dose concentration was verified by serial dilution and plating on TSA. BALB/c mice were challenged via the lateral tail vein with 100 µl of the bacterial suspension two weeks following the second immunization. Two weeks following challenge, the mice were euthanized and kidneys were harvested, homogenized in PBS, and plated on TSA.

### Prosthetic Implant Infection

The prosthetic implant model was conducted in a manner similar to that previously described [43]. Briefly, on the day of challenge, an overnight culture of *S. aureus* SAP149 was centrifuged at 4000×*g* for 10 minutes. The pellet was washed with PBS, counted with a Petroff-Hausser Counting Chamber, and then resuspended to a concentration of 1×10^6^ CFU/ml. Ten C57BL/6 mice per experimental group received implants. Mice were anesthetized via IP injection of 100 mg/kg Ketamine (Ketaset®; Fort Dodge Laboratories, Inc., Fort Dodge, Iowa) and 10 mg/kg Xylazine (Rugby Laboratories, Inc., Rockville Center, NY). The left leg of each mouse was cleansed with povidone iodine and rinsed with 70% ethanol before surgical implantation of an autoclaved 0.25 mm insect pin into the tibia (Fine Science Tools, Foster City, CA) as described [43], [44]. The implanted pin was then inoculated with 1 µl of the *S. aureus* SAP149 suspension. Three weeks following implantation, mice were sacrificed and the left tibiae were harvested, cut into small pieces, and placed in 300 µl of sterile 0.85% saline per 100 µg of bone. Bones were then homogenized using a Polytron PT 1200 handheld homogenizer (Kinematica, Bohemia, NY) at 25,000 rpm, and serial 10-fold dilutions of bone homogenates were plated on TSA II, 5% defbrinated sheep’s blood agar plates to enumerate viable *S. aureus* per gram bone, as previously described [43].

### Statistical Analysis

For all challenge experiments, CFU levels were compared between HlaH35L-immunized animals and adjuvant-only control animals using GraphPad Prism. Differences in CFU levels were evaluated using the untailed Student’s *t* test. For IV challenge experiments, the data from two identical experiments run on separate days were combined. ELISA titers were defined as the ED_50_ for sera pooled within a group from each experiment. ED_50_ values were compared across challenge experiments using ANOVA.

## Results

### HlaH35L Immunization Decreases Disease Severity and Bacterial Colonization in a Skin and Soft Tissue Infection Model of *S. aureus* Infection

**Figure 1:**
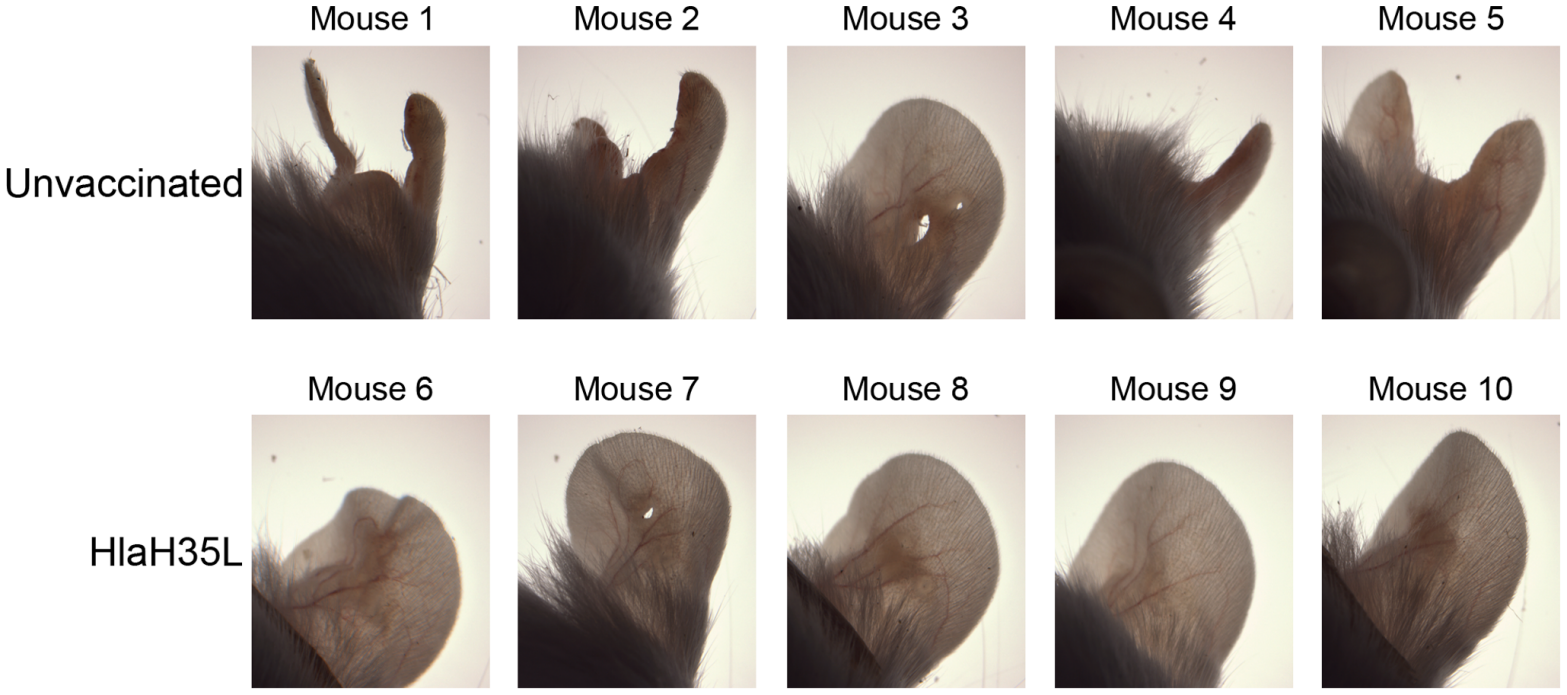
Assessment of tissue damage from SSTI in control vs HlaH35L-immunized mice. Photographs were taken on day 14 post-challenge.

**Figure 2:**
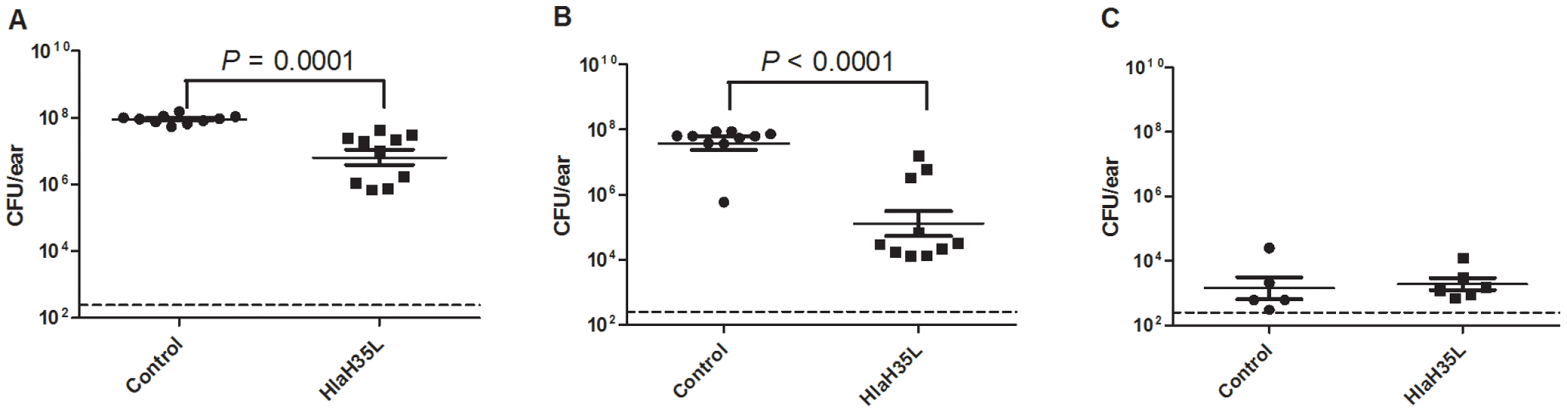
Analysis of HlaH35L in a skin and soft tissue infection model of *S. aureus* infection. Control animals receiving aluminum hydroxide/CpG adjuvant (•) and animals receiving HlaH35L with adjuvant (▪) were compared at days four (A), seven (B), and fourteen (C) post-challenge. The CFU per ear are plotted, with the mean +/− SEM indicated for each group and *P* value calculated by two-tailed Student’s t test. The dashed line indicates the limit of detection.

In order to evaluate the effect of HlaH35L immunization in an SSTI model of *S. aureus* infection, we challenged immunized or control mice with 1×10^9^ CFU *S. aureus* SAP149 in the left ear two weeks after the second immunization. This model was developed by Prabhakara *et al.* [42] and leads to a self-limited, localized infection that generates a necrotic lesion. The challenge was conducted using a Morrow Brown needle such that the bacteria do not penetrate past the upper epidermal layer of the skin. We followed several animals over time in order to monitor lesion formation and subsequent tissue damage (Figure 1). In control animals that received Alhydrogel® and CpG adjuvant without antigen, we saw that mice lost large portions of the affected ears as the infection progressed. However, in HlaH35L-immunized animals, tissue loss was minimal or did not occur (Figure 1). Animals were euthanized at days four and seven post-challenge and serial dilutions of the homogenized, affected ears were plated. Animals receiving HlaH35L with Alhydrogel® and CpG had significantly lower levels of *S. aureus* SAP149 present in the challenged ears than did control mice receiving adjuvant alone at both time points (Figure 2A and 2B). By day 14 post-challenge, all animals had colonization levels approaching the limit of detection (Figure 2C); this result is not unexpected, as this SSTI model is self-limiting [42]. These results were reproducible over two independent experiments. These data indicate that HlaH35L immunization leads to a lesser infection with lower bacterial levels and nominal tissue damage when compared to controls.

### HlaH35L Immunization Decreases Bacterial Colonization of Kidneys in a Systemic Model of *S. aureus* Infection

**Figure 3:**
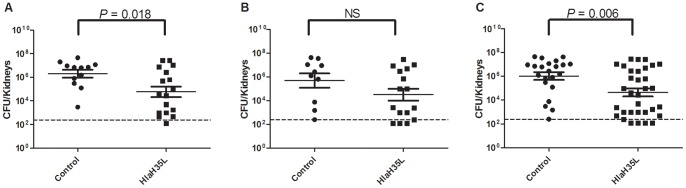
Analysis of HlaH35L in a systemic infection model of *S. aureus* infection. Control animals receiving aluminum hydroxide/CpG adjuvant (•) and animals receiving HlaH35L with adjuvant (▪) were compared in an IV challenge model with 3×10^6^ CFU SAP149 as the challenge dose. Shown are the data for two individual experiments performed identically (A and B) and the combined data (C). The CFU per pair of kidneys are plotted, with the mean +/− SEM indicated for each group and the *P* value determined by two-tailed Student’s t-test. The dashed line indicates the limit of detection.

To test whether HlaH35L immunization lessens *S. aureus* systemic infection, mice were immunized with HlaH35L adjuvanted with Alhydrogel® and CpG, or adjuvant alone as a control. Mice were then challenged with 3×10^6^ CFU of *S. aureus* SAP149 via the lateral tail vein, resulting in bacteria being introduced directly into the bloodstream. When kidneys were harvested two weeks following challenge, we noted a decrease in bacterial levels in the immunized mice. The experiment was run twice; one experiment yielded statistically significant results, whereas the other did not (Figure 3). Combining data from the two experiments to increase effective sample size and therefore increase statistical power demonstrated that immunization with HlaH35L significantly reduced kidney colonization after IV challenge when compared to controls (*P* = 0.006).

### HlaH35L Immunization does not Lessen Bacterial Colonization of Infected Tibiae in a Model of Chronic Biofilm Infection

**Figure 4:**
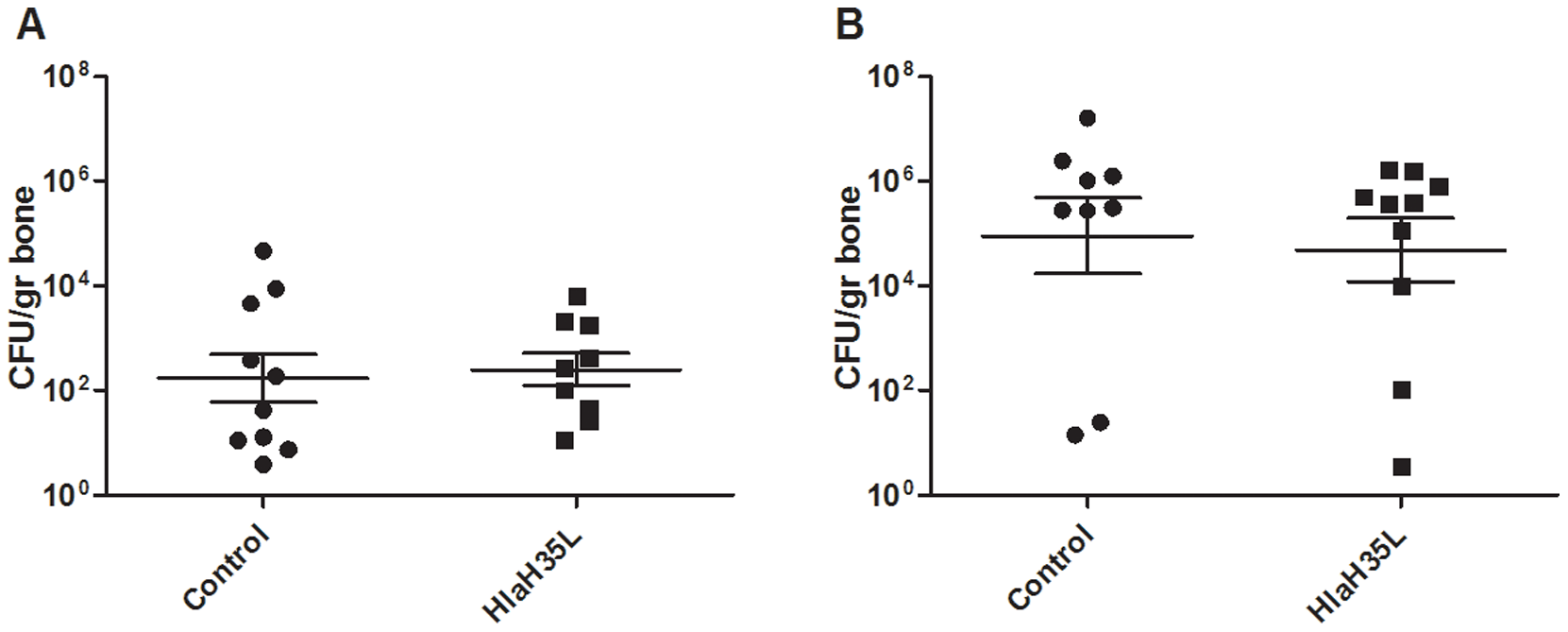
Analysis of HlaH35L in a prosthetic implant model of *S. aureus* infection. Control animals receiving aluminum hydroxide with CpG adjuvant (•) and animals receiving HlaH35L with adjuvant (▪) were compared. Shown are the results for two experiments performed identically (A and B). The CFU per gram of bone are plotted, with the mean +/− SEM indicated for each group.

In order to determine if HlaH35L immunization is able to decrease levels of *S. aureus* in a model of chronic biofilm infection, we challenged immunized and control mice by introducing a metal implant and then inoculating the implant with 1×10^3^ CFU *S. aureus* SAP149. Twenty-one days post-challenge, levels of *S. aureus* in the tibiae of immunized mice were determined and compared to that of mice having received Alhydrogel® and CpG alone. The experiment was performed in duplicate. There was no significant difference in bacterial colonization of affected tibiae observed between groups (Figure 4A and B) in either experiment. These data suggest that HlaH35L immunization does not lessen severity of chronic *S. aureus* biofilm infection.

We should note that this model utilizes C57BL/6 mice instead of BALB/c mice, because BALB/c mice, unlike what is seen in clinical cases of *S. aureus* prosthetic implant infection, can spontaneously clear the infection even if a biofilm is first grown on the pin prior to implantation. In order to determine the importance of mouse strain in the other models, we performed ELISA on pools of sera from immunized mice for each of the three challenge models used iwere anesthetized via IP injection n this study. We found no significant difference between the antibody titers (expressed as ED_50_s) for HlaH35L-immunized animals from the three model groups (data not shown; *P* = 0.874, ANOVA). Moreover, we also performed *S. aureus* SSTI challenge using C57BL/6 mice and found that significantly lower infection levels were obtained in mice immunized with HlaH35L as compared to control mice (data not shown), similar to the results obtained with BALB/c mice. These results indicate that HlaH35L can elicit protection in the skin and soft tissue infection model regardless of whether BALB/c or C57BL/6 mice are used.

## Discussion

While a good deal of research into potential *S. aureus* vaccine candidates has been conducted, the vast majority of publications detailing such investigations have described testing in a single animal model of infection. While an antigen may elicit protection against single, or even similar, disease types, broad protection against the wide variety of infections that a dynamic pathogen such as *S. aureus* causes may be difficult to achieve. Therefore, testing potential candidates in animal models that encompass both acute infections, e.g. skin and soft tissue infections or sepsis, and chronic infections such as osteomyelitis, would be informative.

In this study, we undertook the evaluation of a single protein antigen that has been published in several works to generate a protective response in animals against *S. aureus* infection and that has been included in *S. aureus* vaccines in clinical trials [45]. Inactivated alpha toxin has been shown to reduce bacterial populations and/or protect mice from lethality against pneumonia [32], skin infection [30], and, recently, sepsis [38] and peritoneal abscess models [37]. However, the toxin has not been tested against chronic, biofilm-associated infections. Moreover, those studies used different dosing regimens, adjuvants, and challenge strains. For this work, we eliminated potential laboratory-to-laboratory variability by using the same protein preparation for immunizations and the same challenge strain for protection studies. We also used a single dosing regimen and the same adjuvant for all studies to better mimic how a vaccine would actually be used. We found that HlaH35L combined with Alhydrogel® and CpG administered to mice that were then subjected to *S. aureus* SSTI or systemic challenge led to lower infection levels than did adjuvant alone. The difference between vaccine and control groups was statistically robust in the SSTI model. The difference between the two groups for the systemic infection model, while statistically significant when data from two experiments were combined, was less robust. Possibly, HlaH35L is only marginally protective in our systemic infection model, and the more moderate level of protection required a greater number of animals to reach statistical significance.

We found that the same immunization strategy did not elicit protection in a prosthetic implant infection model. This model was developed by Prabhakara *et al*. to recapitulate what is seen in humans with biofilm infections: The infection remains localized, with bacterial levels constant after seven days. The infection also persists long term (>49 days), and is unable to be cleared by antibiotics or the host immune response [43]. We found that there was no significant difference in the level of *S. aureus* in infected tibiae from either HlaH35L-immunized or control mice. This finding may not be surprising, as microarray analyses have indicated that *hla* is down-regulated during biofilm growth [7], [46]. It is also possible that alpha toxin may be present at some level within the mature biofilm, but that it plays no role in such an environment; alternatively, alpha toxin may not affect biofilm development or bacterial colonization of bone. In these cases, antibodies capable of toxin neutralization would not be expected to be effective in clearing the biofilm-associated bacteria. Regardless of the underlying reason why an HlaH35L vaccine was not protective in this model, this failure indicates that antigens that are chosen based on their effectiveness in other types of staphylococcal infection, such as systemic disease, may not be useful in the case of chronic, biofilm-associated infections.

Because expressed virulence factors vary among different manifestations of *S. aureus* disease, it is important to determine if a vaccine antigen may be broadly protective. To our knowledge, no group has described testing of individual *S. aureus* antigens in both acute and chronic infection models, within a laboratory using the same protein preparation, same dosing regimen, same adjuvant and the same challenge strain. Our work indicates that an antigen that is protective against one disease form may not be protective against other forms. In particular, protection against acute infection may not be predictive of protection against chronic biofilm-associated *S. aureus* disease. These results demonstrate that if a single antigen is to be used, care should be taken to ensure that the antigen is universally expressed, accessible to the host immune response, and necessary for bacterial persistence under all *S. aureus* growth conditions. An alternate strategy might be to formulate a vaccine that includes multiple protective antigens that play critical roles in both acute and chronic forms of infection. In either case, robust testing in multiple animal models may provide useful information regarding the potential of a vaccine to protect against a dynamic pathogen such as *S. aureus*.
